# Putrescine Supplementation Limits the Expansion of *pks*+ *Escherichia coli* and Tumor Development in the Colon

**DOI:** 10.1158/2767-9764.CRC-23-0355

**Published:** 2024-07-22

**Authors:** Manon Oliero, Thibault Cuisiniere, Ayodeji S. Ajayi, Claire Gerkins, Roy Hajjar, Gabriela Fragoso, Annie Calvé, Hervé Vennin Rendos, Annabelle Mathieu-Denoncourt, François Dagbert, Éric De Broux, Rasmy Loungnarath, Frank Schwenter, Herawaty Sebajang, Richard Ratelle, Ramses Wassef, Carole Richard, Marylise Duperthuy, Andrée E. Gravel, Antony T. Vincent, Manuela M. Santos

**Affiliations:** 1 Nutrition and Microbiome Laboratory, Institut du cancer de Montréal, Centre de recherche du Centre hospitalier de l’Université de Montréal (CRCHUM), Montréal, Canada.; 2 Department of Surgery, Faculty of Medicine, Université de Montréal, Montréal, Canada.; 3 Department of Microbiology, Infectiology and Immunology, Faculty of Medicine, Université de Montréal, Montréal, Canada.; 4 Digestive Surgery Service, Department of Surgery, Centre hospitalier de l’Université de Montréal (CHUM), Montréal, Canada.; 5 Drug Discovery Platform, Research Institute McGill University Health Centre, Montreal, Canada.; 6 Département des sciences animales, Faculté des sciences de l’agriculture et de l’alimentation, Université Laval, Quebec City, Canada.; 7 Institut de biologie intégrative et des systèmes, Université Laval, Quebec City, Canada.; 8 Department of Medicine, Faculty of Medicine, Université de Montréal, Montréal, Canada.

## Abstract

*Escherichia coli* that harbor the polyketide synthase (*pks*) genomic island produce colibactin and are associated with sporadic colorectal cancer development. Given the considerable prevalence of *pks+* bacteria in healthy individuals, we sought to identify strategies to limit the growth and expansion of *pks+ E. coli*. We found that culture supernatants of the probiotic strain *E. coli* Nissle 1917 were able to inhibit the growth of the murine pathogenic strain *pks+ E. coli* NC101 (EcNC101). We performed a nontargeted analysis of the metabolome in supernatants from several *E. coli* strains and identified putrescine as a potential postbiotic capable of suppressing EcNC101 growth *in vitro*. The effect of putrescine supplementation was then evaluated in the azoxymethane/dextran sulfate sodium mouse model of colorectal cancer in mice colonized with EcNC101. Putrescine supplementation inhibited the growth of *pks+ E. coli*, reduced the number and size of colonic tumors, and downmodulated the release of inflammatory cytokines in the colonic lumen. Additionally, putrescine supplementation led to shifts in the composition and function of gut microbiota, characterized by an increase in the *Firmicutes*/*Bacteroidetes* ratio and enhanced acetate production. The effect of putrescine was further confirmed *in vitro* using a *pks+ E. coli* strain isolated from a patient with colorectal cancer. These results suggest that probiotic-derived metabolites can be used as an alternative to live bacteria in individuals at risk of developing colorectal cancer due to the presence of *pks+* bacteria in their colon.

**Significance:**

Putrescine supplementation inhibits the growth of cancer-promoting bacteria in the gut, lowers inflammation, and reduces colon cancer development. The consumption of healthy foods rich in putrescine may be a potential prophylactic approach for individuals at risk of developing colorectal cancer due to the presence of *pks+* bacteria in their colon.

## Introduction

*Escherichia coli* and other members of *Enterobacteriaceae* that harbor the polyketide synthase (*pks*) island are part of the pool of pathobionts involved in the onset and development of colorectal cancer (1). *pks*+ bacteria produce colibactin, a genotoxin that forms DNA adducts, leading to interstrand cross-links and double-strand breaks (DSB) that induce DNA mutations in mammalian cells and enhance intestinal tumorigenicity in the azoxymethane (AOM)/dextran sulfate sodium (DSS) murine model (2). Up to 68% of patients with colorectal cancer are colonized by *pks+ E. coli* (3), and the presence of the phylogroup of *pks+ E. coli* is increasing worldwide (4, 5). Given the causal link between *pks+ E. coli* and sporadic colorectal cancer development, microbiota-targeted therapies are being considered to limit the presence of *pks+ E. coli* and reduce the risk of colorectal cancer.

The probiotic strain *E. coli* Nissle 1917 (EcN), discovered during World War I to prevent *Shigella*-induced diarrhea, has been used as a probiotic in the relief and management of gastrointestinal disorders (6). Despite demonstrating probiotic properties (7) and innocuity in infants (8), EcN harbors the *pks* genomic island and releases colibactin, suggesting that the live form of EcN is potentially harmful (9). Recently, inactivated forms of EcN have been proven to retain probiotic properties by attenuating colitis in the DSS mouse model (10) and by reducing the proliferation of HT-29 colon adenocarcinoma cells (11). Furthermore, we previously demonstrated that pasteurized EcN lessened the carcinogenic activity of the murine *pks+ E. coli* strain NC101 (EcNC101; ref. 12).

Given the high prevalence of colibactin-producing bacteria in the general population and patients with colorectal cancer (3), we aimed to find potential postbiotics derived from EcN to safely lower the expansion of *pks+ E. coli* and/or colibactin production. We first performed a nontargeted analysis of the metabolome of these *E. coli* strains and identified putrescine as a potential postbiotic. We used the AOM/DSS mouse model of colorectal cancer colonized with EcNC101 to evaluate the effect of putrescine on tumor development and gut microbiota composition. Growth inhibition by putrescine was also tested on a genotoxic strain of *pks+ E. coli* isolated from a patient with colorectal cancer.

## Materials and Methods

### Bacterial strains

*E. coli* strains in this study included the pathogenic murine strain EcNC101 wild-type (WT), the EcNC101 depleted for the gene *colibactin P* (*ΔclbP*; both EcNC101 strains were a gift from Dr. Christian Jobin, Cancer Microbiota & Host Response, UF Health Cancer Center, University of Florida), the probiotic EcN (Mutaflor, Pharma-Zentrale GmbH, Germany), and *E. coli* K12 (EcK12; ER2738, New England Biolabs Ltd., Whitby, ON, Canada). All strains of *E. coli* were grown from glycerol stocks in lysogeny broth (LB) at 37°C, shaking at 150 revolutions per minute (rpm) overnight, and subcultured in appropriate media.

### Bacterial growth

*E. coli* strains were subcultured at 1/100 dilution in M9. For growth experiments, 1 × 10^7^ colony-forming units (CFU)/100 μL of bacterial cells were inoculated in a transparent 96-well plate (Sarstedt, Nümbrecht, Germany) with putrescine (51799; Sigma-Aldrich) and were grown with shaking at 37°C. Bacterial growth (OD_600nm_) was recorded every hour using a multimode microplate reader.

### Bacterial competition assay

To measure EcNC101 growth compared with EcN growth, we transformed strains with the plasmids pUCP20T-E2Crimson and pUCP20T-morange (gifts from Mariette Barbier, Department of Microbiology, Immunology, and Cell Biology, West Virginia University, Morgantown, WV, USA; Addgene plasmid #78473; http://n2t.net/addgene:78473; RRID: Addgene 78473/Addgene plasmid #78468; http://n2t.net/addgene:78468; RRID: Addgene_78468) to distinguish them by crimson and orange fluorescence, respectively (13). The strains were grown overnight in LB medium supplemented with ampicillin. Each competition between two strains was compared with the growth of each strain grown alone (control) in parallel with the competition assay. The competitor strains were inoculated at 10^7^ CFU/mL in a starting volume of 5 mL LB and grown at 37°C for 24 hours without shaking. After 1 day, 50 μL of the competitor strain culture was inoculated in fresh LB (1/100) and grown for 24 hours, and this was repeated for 3 days. Fluorescence was recorded every day from the starting point in a Spark multimode microplate reader (Tecan Group Ltd.).

### Bacterial arginine assay

*E. coli* strains were inoculated at 1 × 10^7^ CFU/100 μL in minimal medium (M9) supplemented with 1 mmol/L L-arginine and 0.015 g/L bromocresol purple (Bio Basic Inc., Markham, ON, Canada) under anaerobic conditions for 32 hours. At 4, 8, 16, and 32 hours, culture aliquots were taken and centrifuged at 10,000 rpm for 1 minute. Culture aliquots of 100 μL were then plated in a transparent 96-well plate (Sarstedt, Nümbrecht, Germany), and optical density (OD) at 560 nm (Purple, alkaline pH, and ornithine fermentation) was read in a Spark multimode microplate reader (Tecan Group Ltd., Quebec, Canada).

### Nuclear magnetic resonance

*E. coli* strains were seeded at 10^7^ CFUs in LB at 37°C at 150 rpm for 7 hours under anaerobic conditions. The cultures were centrifuged at 12,000 rpm for 2 minutes. Supernatants were harvested, filtered (2 μm), and stored at −80°C until further analysis. Before analysis, 50 μL of a stock solution of sodium (3-trimethylsilyl)-2, 2, 3, 3-tetradeuteriopropionate (TSP)/D_2_O was added, making a final concentration of 0.5 mmol/L in TSP to be used as a chemical shift reference at 0.0 ppm. The supernatant (450 μL) was transferred into a 5-mm high-resolution nuclear magnetic resonance (NMR) tube for ^1^H NMR spectroscopic analysis. NMR experiments were performed at 298 K on an autosampler Avance III HD 600 MHz Bruker spectrometer (Bruker, Montreal, Quebec, Canada) equipped with BBI 5-mm z-gradient probe. Standard one-dimensional ^1^H NMR spectra were acquired (Bruker pulse sequence noesygppr1d) using a relaxation delay and a mixing time of 4 seconds and 10 ms, respectively, for a 90° pulse width of 7.6 μs. A total of 65,536 complex data points were accumulated for 32 scans over a spectral width of 12,019 Hz using an acquisition time of 2.73 seconds. The free induction decays were then apodized with an exponential function, resulting in a line broadening of 0.3 Hz and zero filled to 131 k data points before the Fourier transform. The data were automatically phased, the baseline was corrected, and the spectra were referenced to the internal standard (TSP; δ 0.0 ppm) using TopSpin 4.3.1 (Bruker BioSpin, Germany).

### LC-MS/MS

*E. coli* strains were seeded at 10^7^ CFUs in M9 supplemented with 0.25, 0.5, and 1 mmol/L of L-arginine (Sigma-Aldrich Canada Co, Oakville, ON, Canada) and grown at 37°C at 150 rpm for 7 hours under anaerobic conditions. The cultures were centrifuged at 12,000 rpm for 2 minutes, and the supernatants were harvested, filtered (0.2 μm), and stored at −80°C. For LC-MS/MS analysis, 80 μL of water and 900 μL of 90% acetonitrile in water with 3.3 μmol/L of d5-glutamine (used as internal standard) were added to 20 μL of the supernatant or putrescine standards, and the mixture was mixed (10 minutes, 4°C) and centrifuged (20,000 *g*, 10 minutes, 4°C). Chromatographic separation of samples of 2 μL was performed on a Poroshell 120 HILIC-Z, 2.1 × 100 mm, 2.7 μm particles HPLC column (Agilent Technologies) at 0.8 mL/minutes and 30°C using two mobile phases A (20 mmol/L ammonium formate in water) and B (20 mmol/L ammonium formate in 90% acetonitrile in water) in the following gradient elution: 0 minutes 100% B, 10 minutes 70% B, and 11 minutes 100% B. Analytes were detected in positive ion mode highlighting the following transitions: 88.94/72.20; d5-Gln: 152.0/89.0; ornithine: 133.0/70.1; arginine: 175.1/70.2.

### Animal experiments

Experiments were performed according to the guidelines of the Canadian Council on Animal Care and were approved by the Institutional Animal Care Committee of the Centre de recherche du Centre hospitalier de l’Université de Montréal (CRCHUM). C57BL/6 mice were originally obtained from The Jackson Laboratory and bred at our animal facility under specific pathogen–free conditions. Female mice of 6- to 8-week-old were used at the beginning of the experiments and were maintained under standard 12:12 light/dark conditions. They were cohoused at 2 to 3 mice per cage and were allowed *ad libitum* access to food and water. Additionally, the experiment was conducted in batches, with mice randomly assigned to one of the four experimental groups to avoid any bias until obtaining nine mice in each group. Mice received a standard diet (Envigo Teklad Diets, TD2918) during the entire experiment. The AOM (A5486; Sigma-Aldrich)/DSS (DB001; TdB Labs, Uppsala, Sweden) model was induced by intraperitoneal injection of 15 mg/kg of AOM in 18 to 22 g female mice. A week after the AOM injection, mice were treated with an antibiotic cocktail [ampicillin (0.25 mg/mL) and streptomycin (0.75 mg/mL) and colistin (1 mg/mL)] in drinking water for 3 days. A day after the end of the antibiotic treatment, mice received an oral gavage of 200 μL of appropriate bacterial suspension (10^8^ CFUs) in sterile sodium chloride (0.9%). Mice were then subjected to three cycles of 2% to 2.5% DSS for 5 days, followed by a recovery period of 14 days. In parallel, mice were treated with 1% dihydrochloride putrescine (P5780; Sigma-Aldrich) or 1% hydrochloride in the drinking water. At the end of the experiment (day 69), mice were anesthetized with an intraperitoneal injection of sodium pentobarbital and killed by cervical dislocation. Colons were measured and then cut in the longitudinal part. Two researchers, blinded to the groups, separately counted the number of tumors and measured tumor size.

### IHC

Formalin-fixed, paraffin-embedded sections of colonic tissue were stained using the BenchMark XT autostainer (Ventana Medical Systems, Tucson, AZ, United States). IHC staining was carried out on frozen sections using specific antibodies anti-Ki67 (Biocare CRM325A, Biocare Medical, Pacheco, CA, United States) and the anti–β-catenin (ab32572; Sigma-Aldrich). Reactions were performed using the iView DAB detection kit, and counterstaining was achieved with hematoxylin and bluing reagents at 1/150 dilution.

### Fecal water, protein quantification, and ELISA

Fecal samples were reconstituted in phosphate-buffered sodium (PBS) containing 0.1% Tween 20 at a concentration of 25 mg feces/mL and vortexed for 5 minutes to yield a homogeneous suspension. The upper aqueous portion was collected by centrifugation (12,000 × *g* for 10 minutes at 4°C) and stored at −20°C until analysis. Protein quantification from fecal samples was carried out using Pierce BCA protein assay kit (Thermo Fisher Scientific). The levels of lipocalin 2 (Lcn-2), IL6, TNFα, and IL10 were quantified in fecal water using the Human or Mouse Lipocalin-2/NGAL ELISA kit (R&D Systems, Minneapolis, MN) and the ELISA MAX Standard Set Mouse IL6, TNFα, and IL10 (BioLegend, San Diego, CA, USA; Cerdalane distributor). The plates were read on a multimode microplate reader.

### Analysis of 16S rRNA sequencing

Bacterial DNA was extracted and 16S ribosomal RNA (rRNA) library preparation and sequencing that targeted the V5–V6 region (primers: P609D and P699R) of the 16S rRNA gene were performed, followed by the DADA2 pipeline, as previously described (14). An average of 36,227 (± 1151 SEM) high-quality 16S rRNA sequences were generated per sample. Amplicon sequence variants were assigned taxonomy using the Silva training set v138.1 (15). Alpha-diversity and β-diversity were computed using the phyloseq package (version 1.32.0). Statistical significance of distances between groups was performed using the Adonis function from the vegan R package (version 2.5, RRID: SCR_011950). Metabolic pathway inference was performed using the Tax4Fun2 R package with default parameters (version 1.1.6; ref. 16).

### RT-PCR

RT-PCR was performed using PowerUp SYBR Green master mix (Thermo Fisher Scientific) and the RG 3000A R (Qiagen, Québec, Canada). Primers used in the study are presented in Supplementary Table S1. Relative quantitation was performed using standard curves constructed from serial dilutions of PCR products. Expression levels of targeted genes were normalized to 16S rRNA.

### Short-chain fatty acids quantification

Quantification of short-chain fatty acids (SCFA) was performed using electrospray ionization mass spectrometry at the Metabolomics Core Facility of CRCHUM, as previously reported (17).

### Patient recruitment and sample collection

Human studies were conducted in accordance with the Declaration of Helsinki and the Belmont Report and approved by the Research Ethics Board of the Centre hospitalier de l’Université de Montréal (numbers: 19.021, 21.359, 21.153, and 21.368). All participants provided written informed consent prior to samples and data acquisition. Individuals with inflammatory bowel disease, polyps, or antibiotic treatment 6 months before the sampling were excluded from the control group. Participants were requested to provide a fresh fecal sample collected at home following the International Human Microbiome Standards procedure. Samples were collected in hermetic containers with an anaerobic sachet (BD BBL GasPak anaerobic indicator, BD, ON, Canada), and aliquots of 20 mg were stored at −80°C for analysis.

### Isolation and identification of *pks+ E. coli* in fecal samples

Fecal samples were reconstituted in sodium chloride (0.9%) at a concentration of 20 mg feces/mL and vortexed for 5 minutes to yield a homogeneous suspension. The fecal suspension (100 μL) was plated on MacConkey agar (Thermo Scientific Oxoid), and Gram-negative lactose-fermenting strains of *E. coli*, *Enterobacteria*, and *Klebsiella* were quantified based on the number of red colonies. Three red colonies from the MacConkey culture were selected randomly and isolated on tryptic soy agar plates and cultured aerobically at 37°C for 24 hours. Identification of bacteria was performed using a matrix-assisted laser desorption/ionization–time-of-flight technique, and the presence of the *colibactin A* gene was confirmed by PCR. Harvested colonies were diluted in 200 μL of sterile water and boiled for 10 minutes. Simultaneous amplification of *colibactin A* and *E. coli* 16S rRNA genes was performed using PowerUp SYBR Green master mix (Thermo Fisher Scientific, Waltham, MA, USA) in the RG 3000A R PCR machine (Qiagen Inc.; ref. 3). Whole-genome sequencing was carried out on an Illumina NovaSeq apparatus at SeqCenter (Pittsburg, USA). Sequencing reads were filtered using fastp version 0.23.2 (18) *de novo* and assembled into contiguous sequences using shovill version 1.1.0. Sequence annotation and serotype determination were performed using Bakta version 1.8.1 (19) and the SeroTypeFinder web server (20), respectively. The genomic sequences closest to those of the CRCmo10 strain were determined by calculating the Jaccard similarity percentage and average nucleotide identity against the RefSeq database using SourMash version 4.8.2 (RRID: SCR_024347).

### THP1 cell culture

Luciferase THP1-Lucia NF-κB cells enable monitoring of NF-κB signal transduction pathways by measuring luciferase and were purchased from InvivoGen (Cerdalane distributor, Burlington, Ontario, Canada) in January 2022. This monocyte human cell line was tested for *Mycoplasma* contamination on the 7-day culture of the last passage using a universal mycoplasma PCR detection kit (abm, Richmond, British Columbia, Canada). Cells were authenticated by examination of morphology and production of luciferase. Individual cryovials were thawed, and cultures did not exceed 20 passages. Cells were grown in RPMI medium supplemented with 10% heat-inactivated FBS and maintained in 75-cm^2^ culture flasks at 37°C in a 5% CO_2_ (v/v) incubator in a humidified atmosphere. Cells were then dispensed (4 × 10^5^ cells/well) in a 24-well tissue culture plate with RPMI supplemented with 1 μL/mL lipopolysaccharides (LPS; L6529; Sigma-Aldrich) and 0.25, 0.5, and 1 mmol/L of putrescine. After 24 hours of culture, an aliquot was taken, and 25 μL of QUANTI-Luc (InvivoGen, Cerdalane distributor) was added to the well. Luciferase was measured using a multiplate reader. Cells were manually counted with blue trypan dye, and the results were expressed in count per second divided by cell density.

### HT-29 cell culture

The human colonic adenocarcinoma cell line HT-29 (ATCC HTB-38; RRID: CVCL_0320) was a gift from Dr. Petronela Ancuta, (Department of Microbiology and Immunology, Université de Montréal) and was authenticated using short tandem repeat profiling in October 2022 and by examination of morphology and proliferation *in vitro*. The primary human tumor cell line was tested for Mycoplasma contamination on a 7-day culture of the last passage using a universal mycoplasma PCR detection kit (abm). Individual cryovials were thawed and cells used between the 15th to 25th passage. Cells were grown in RPMI medium supplemented with 10% FBS (Thermo Fisher Scientific) and maintained in 75-cm^2^ culture flasks at 37°C in a 5% CO_2_ (v/v) incubator in a humidified atmosphere. Briefly, the *E. coli* moCRC10 strain from glycerol stocks was grown in LB at 37°C, and HT-29 cells were dispensed (4 × 10^5^ cells/well) in a 24-well tissue culture plate (Falcon) for megalocytosis assays. For in-cell Western assay, cells were dispensed (1 × 10^5^ cells/well) in a black 96-well plate (Greiner Bio-One) at 37°C in a 5% CO_2_ atmosphere. After 1 day, HT-29 cells were infected at a multiplicity of infection (MOI) of 0.5, 1, 5, and 10 with *E. coli* moCRC10. After 4 hours of infection, cells were washed and incubated in the cell culture medium supplemented with 200 μg/mL gentamicin (VWR).

### Megalocytosis assay

At 72 hours postbacterial infection, cells were fixed with 4% paraformaldehyde (Thermo Fisher Scientific) for 15 minutes, washed, and stained with 1 mmol/L methylene blue (Sigma-Aldrich). Pictures were taken under a Nikon Eclipse TE300 microscope (Nikon Healthcare, Québec, Canada), and images were acquired using NIS-Elements BR4.00.03 software (200× magnification). Methylene blue extraction solution was used to quantify cell damage and megalocytosis at 660 nm absorbance (multimode microplate reader).

### Fluorescent immunostaining of γ-H2AX by in-cell Western assay

Quantification of DNA DSBs was performed using the in-cell Western assay, as described previously (21). Following bacterial infection, cells were fixed (4% paraformaldehyde), permeabilized, blocked, and then incubated for 2 hours with rabbit monoclonal anti–γ-H2AX (BioLabs) at 1/200 dilution. The secondary antibody IRDye 800CW goat anti-rabbit (Biotium, Wisconsin, United States) was applied simultaneously with 1/500 dilution of RedDot2 (Biotium) for DNA labeling. The DNA and γ-H2AX were visualized using an Odyssey infrared imaging scanner (LI-COR model 9120, Québec, Canada) with red denoting RedDot2 and green for IRDye 800CW goat anti-rabbit. Images were processed using Image Studio (Version 3.1).

### Statistics

All data were analyzed using GraphPad Prism (Version 5.0, GraphPad software, San Diego, CA, USA). When the data did not pass the Shapiro–Wilk normality test, log(Y) transformation was applied to the data. *P* values < 0.05 were considered statistically significant. For associations between variables, Spearman correlation was computed. Differential abundance analysis of the gut microbiota composition and function was performed using DESeq2 and linear discriminant analysis effect size, respectively.

### Data availability

The data generated in this study are available within the article and its supplementary data files. This whole-genome shotgun sequences of strain CRCmo10 have been deposited at DDBJ/ENA/GenBank under the accession JAUKUY000000000, and 16S rRNA data generated in this study are publicly available in the NCBI SRA repository (PRJNA1011886).

## Results

### Putrescine produced by the probiotic EcN inhibits the growth of EcNC101

**Figure 1:**
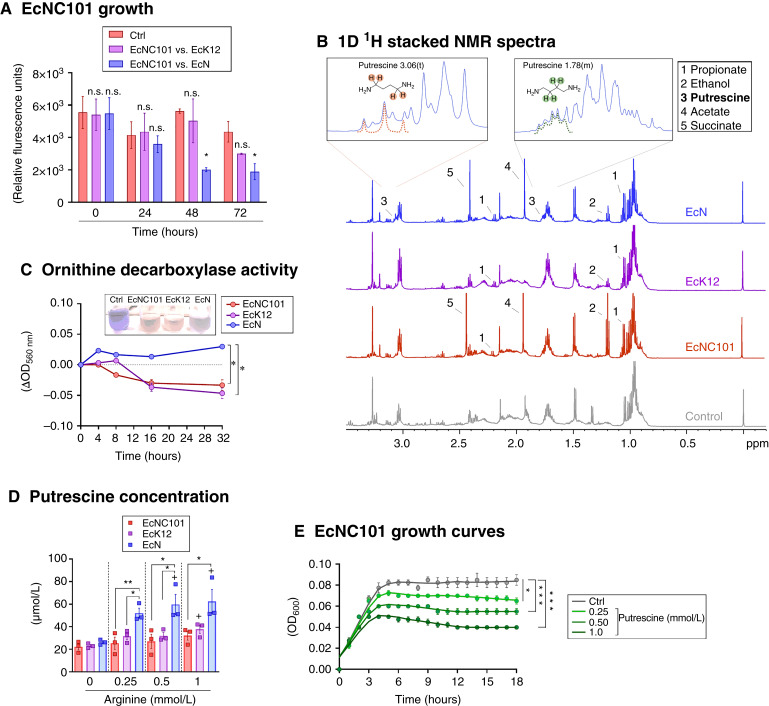
Putrescine inhibits the growth of EcNC101. **A,** Growth of EcNC101 in liquid competition assays with K12 or EcN strains in comparison with the growth of EcNC101 without competition (Ctrl) at each time point (ANOVA, compared with single strain culture of EcNC101 at each time point; *N* = 4). **B,** Representative one-dimensional ^1^H NMR spectra (−0.2–3.5 ppm) of supernatants from EcN, EcK12, EcNC101, and control (LB); sodium (TSP) at 0.0 ppm was the reference peak. Inset images represent putrescine peaks by ^1^H NMR. **C,** ODC activity of EcNC101, EcK12, and EcN in minimal medium (M9) with arginine (repeated-measure ANOVA; *N* = 4). Representative image of the culture media change of color at 32 hours. **D,** Putrescine concentration measured in the supernatant of EcNC101, EcK12, and EcN grown in M9 in the absence or presence of an increasing concentration of arginine for 7 hours [ANOVA, compared with control (0 mmol/L arginine) of each strain; *N* = 3]. **E,** Growth curves of EcNC101 in M9 in the absence or presence of putrescine supplemented at different concentrations (ANOVA, compared with control; *N* = 4). n.s., nonsignificant; *, *P* < 0.05; **, *P* < 0.01; ***, *P* < 0.001.

We previously showed that the probiotic EcN inhibited the growth of EcNC101 both *in vitro* and in *Apc*^*Min/+*^ mice (12). Here, we confirmed the inhibitory effect of EcN in competition with EcNC101. The control strain of EcK12 failed to compete with EcNC101 (Fig. 1A). EcN, on the other hand, was able to inhibit EcNC101 growth after 48 hours of coculture (Fig. 1A).

To identify potential metabolites responsible for inhibiting the growth of EcNC101, we performed a nontargeted analysis of the metabolome in culture supernatants of the strains EcNC101, EcK12, and EcN. A typical ^1^H NMR spectrum acquired from the supernatant of *E. coli* strains is shown in Fig. 1B and Supplementary Fig. S1A. The main metabolites in the NMR spectra were identified according to the Human Metabolome Database (22). All the strains of *E. coli* produced propionate and ethanol; however, succinate and acetate were only detected in the supernatants of EcN and EcNC101. Most importantly, only EcN produced detectable amounts of putrescine (Fig. 1B; inset image).

The capacity of EcN to generate putrescine was subsequently validated by assessing the activity of the ornithine decarboxylase (ODC) enzyme, responsible for the conversion of arginine to ornithine and then to putrescine (Supplementary Fig. S1B; ref. 23). The activity of the ODC, measured by the alkalization of the medium (OD_560nm_; ref. 24), was significantly higher in EcN compared with EcNC101 and EcK12 (Fig. 1C), and only EcN consumed arginine to produce ornithine (Supplementary Fig. S1C). Accordingly, when the minimal medium (M9) was supplemented with 0.25 mmol/L of arginine, EcN released a significantly higher amount of putrescine compared with EcK12 (1.65-fold, *P* < 0.031) and EcNC101 (2.05-fold, *P* < 0.009; Fig. 1D).

We next assessed the effect of putrescine supplementation on EcNC101 growth. As shown in Fig. 1E, adding putrescine to M9 at concentrations ranging from 0.25 to 1 mmol/L significantly reduced bacterial growth in a concentration-dependent manner. These data suggest that *in vitro*, the polyamine putrescine secreted by EcN lowers EcNC101 growth. These results do not exclude the possibility that the observed effects on bacterial growth may result from additional growth inhibitors being secreted by EcN. Nonetheless, given the ability of putrescine to inhibit the growth of cancer-promoting EcNC101 *in vitro*, we next set out to test its effects *in vivo* using a preclinical colorectal mouse model.

### Putrescine supplementation lowers EcNC101 colonization and tumor development in the AOM/DSS mouse model of colorectal cancer

**Figure 2:**
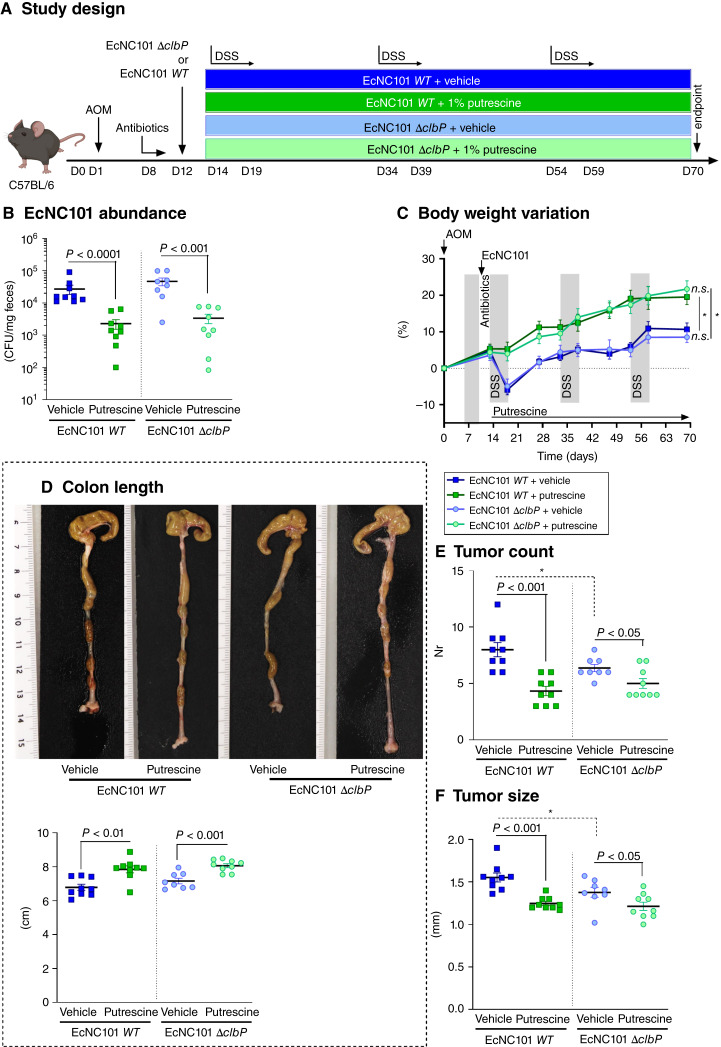
Putrescine supplementation lowers EcNC101 colonization and tumor development in the AOM/DSS colorectal cancer mouse model. **A,** Study design. **B,** EcNC101 abundance at the endpoint was assessed by CFUs (log-scale representation). **C,** Body weight variation of mice throughout the experiment (mean ± SEM; repeated-measure ANOVA, *post hoc* Tukey; *, *P* < 0.01, between the groups). **D,** Representative pictures of the colon and quantification of the colon length. Colonic (**E**) tumor count and (**F**) tumor size. ANOVA, *post hoc* Tukey; *, *P* < 0.05, between EcNC101 *WT* and EcNC101 *ΔclbP* of the same treatment group; *N* = 8–9 per group.

To investigate the effect of putrescine on tumor development in mice colonized with EcNC101, C57BL/6 mice were treated with AOM and were then divided into two groups: Group 1 was infected with the EcNC101 WT, whereas group 2 was colonized with the colibactin-deficient mutant strain EcNC101 (*ΔclbP*; ref. 25). Two days after the bacterial infection, each group was further divided into two: One was given a vehicle as a control, and the other was given putrescine in parallel with the three DSS cycles (Fig. 2A).

EcNC101 abundance was quantified by culturing fecal homogenates on MacConkey agar plates and counting CFUs. At the end of the experiment, EcNC101 was detected at lower concentrations in fecal samples of mice treated with putrescine compared with those of mice given the vehicle alone, independently of the ability to produce colibactin (Fig. 2B). These results indicate that any differences observed later among treatment groups are not due to variations in colonization between EcNC101 WT and *ΔclbP*.

Body weight progress was similar in all four experimental groups until day 14, after which mice supplemented with putrescine gained more weight than control mice, indicating less severity of the disease. Additionally, unlike control mice, putrescine-treated mice did not lose weight after the first DSS cycle. Mice colonized with EcNC101 WT or *ΔclbP* mutant showed similar body weight within the treatment group or the control group (Fig. 2C). Colon shortening, a parameter of DSS-induced epithelium injury (26), had significantly improved in mice that received putrescine compared with vehicle-treated mice (Fig. 2D).

As expected from the AOM/DSS model, tumors in the digestive tract were present only in the colon. Mice colonized with EcNC101 WT had more tumors (Fig. 2E) that were larger in size (Fig. 2F) compared with mice colonized with EcNC101 *ΔclbP*, as determined by counting colonic tumors (vehicle-treated mice: WT 8.0 ± 0.6 vs. *ΔclbP* 6.4 ± 0.3 tumors) and measuring tumor size (vehicle-treated mice: WT 1.55 ± 0.05 vs. *ΔclbP* 1.38 ± 0.06 mm). This effect was attributed to the presence of colibactin, as expected (27). Mice supplemented with putrescine had lower tumor counts and size compared with those treated with vehicle in both EcNC101 WT and EcNC101 *ΔclbP* colonized groups. Notably, Ki67 staining, a marker of cell proliferation, was higher in mice colonized with EcNC101 WT compared with those colonized with EcNC101 *ΔclbP*, indicating increased uncontrolled proliferation associated with colorectal cancer development. Interestingly, mice supplemented with putrescine exhibited increased Ki67 levels compared with vehicle-treated mice in the EcNC101 *ΔclbP* colonized groups, which suggests a potential stimulatory effect of putrescine on cell renewal that is further confirmed by elevated β-catenin levels in those mice (Supplementary Fig. S2).

### Putrescine lowers inflammation and limits EcNC101 expansion in the colon

**Figure 3:**
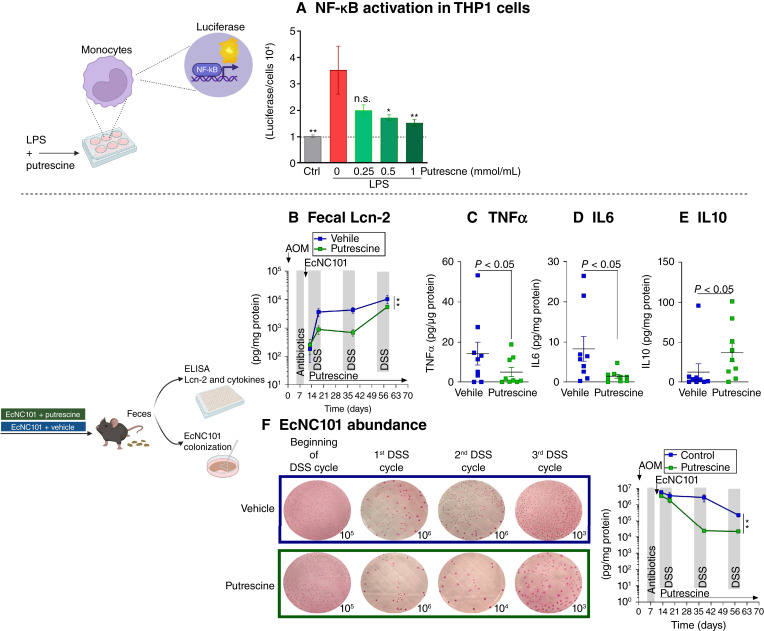
Putrescine decreases EcNC101 expansion and is associated with lower inflammation in mice. **A,** NF-κB activation in THP1 cells treated with *E. coli* LPS (1 μL/mL) and putrescine (*N* = 4; mean ± SEM; ANOVA, *post hoc* Dunnett compared with cells treated with LPS and 0 mmol/L/mL putrescine). **B,** Fecal Lcn-2 after DSS-induced flares (mean ± SEM, repeated-measure ANOVA). **C,** Fecal IL6, (**D**) TNFα, and (**E**) IL10 levels at day 56 (Student *t* test); n.s., nonsignificant; *, *P* < 0.05; **, *P* < 0.01. **F,** EcNC101 CFUs after DSS-induced flares (mean ± SEM, repeated-measure ANOVA). *N* = 9 per group.

Inflammation is a hallmark of the AOM/DSS mouse model caused by repeated DSS cycles (28). Using the human leukemia monocytic cell line THP1-Lucia, which expresses luciferase under the control of the NF-κB promoter, we found that putrescine can decrease inflammation via NF-κB inhibition (Fig. 3A).

We then evaluated the effect of putrescine on inflammation after each DSS cycle in the mice. The fecal level was assessed in mice colonized with EcNC101 WT. Compared with mice receiving vehicle, mice given putrescine presented significantly lower levels of fecal Lcn-2, a sensitive marker of inflammation in patients with inflammatory bowel diseases (vehicle: 23 × 10^4^ vs. putrescine: 8 × 10^4^ AUC; Fig. 3B; ref. 29) through the three DSS cycles. In addition, fecal levels of the proinflammatory cytokines TNFα and IL6 were assessed following the last DSS cycle (Fig. 3C and D) and were significantly lower in putrescine-treated mice (TNFα: vehicle 14.3 ± 5.7 vs. putrescine 5.1 ± 2.4 pg/μg; and IL6: vehicle 8.3 ± 3.1 vs. putrescine 1.4 ± 0.5 pg/μg). Conversely, the levels of the anti-inflammatory cytokine IL10 (Fig. 3E) were higher in mice given putrescine compared with vehicle-treated animals (vehicle: 12.8 ± 10.4 vs. putrescine: 37.2 ± 11.5 pg/μg).

A proinflammatory gut environment has been shown to favor the growth of bacteria from the *Enterobacteriaceae* family which, along with many harmless symbionts, includes numerous pathogens (30)*.* Therefore, we followed EcNC101 levels after DSS-induced flares by culturing fecal homogenates on MacConkey agar plates and counting CFUs. Putrescine supplementation significantly reduced the expansion of EcNC101 during flares (vehicle: 12 × 10^7^ vs. putrescine: 3 × 10^7^ AUC; Fig. 3F). Taken together, these results show that putrescine inhibits inflammation and EcNC101 expansion.

### Putrescine induces shifts in the gut microbial community

To assess the effects of putrescine supplementation on the gut microbiota composition, 16S rRNA amplicon sequencing of DNA extracted from murine fecal samples before (D0) and after (D70) putrescine treatment was analyzed.

**Figure 4:**
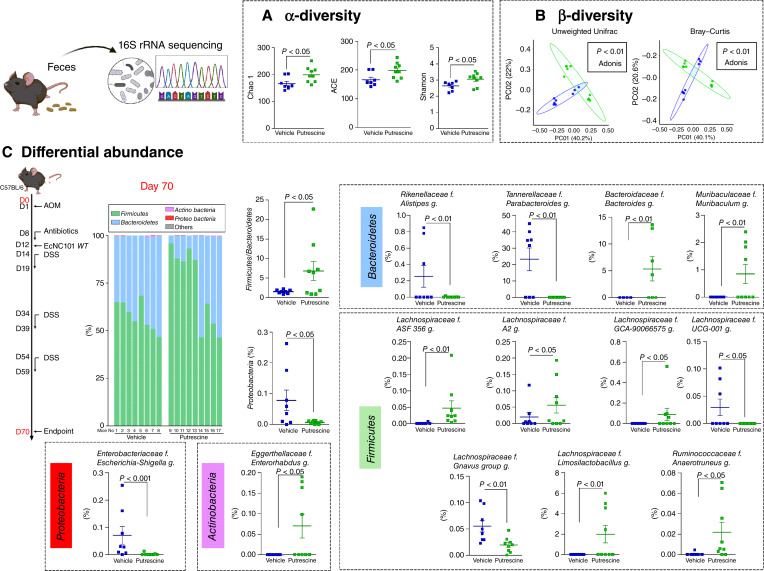
Gut microbiota modulation by putrescine supplementation in the AOM/DSS mouse model of colitis-associated colorectal CRC. **A,** Chao1, ACE, and Shannon indexes at day 70 (mean ± SEM, *t* test). **B,** Principal coordinate analysis (PCoA) of the unweighted UniFrac and Bray–Curtis distances. **C,** Differential bacterial abundance at the phylum and genus levels (mean ± SEM, FDR corrected). Bar graphs of relative abundances at the phylum level at day 70. Each stacked bar represents a single mouse. *N* = 8–9 mice per group.

Alpha-diversity indexes Chao1, abundance-based coverage estimator (ACE), and Shannon were computed. As shown in Fig. 4A, Chao1 (vehicle: 167 ± 23 vs. putrescine: 200 ± 28, *P* < 0.05), ACE (vehicle: 167 ± 8 vs. putrescine: 198 ± 9, *P* < 0.05), and Shannon (vehicle: 2.64 ± 0.09 vs. putrescine: 3.06 ± 0.13, *P* < 0.05) indexes at D70, but not at D0 (Supplementary Fig. S3A), were significantly higher when mice were given putrescine.

Analysis of the level of differences between experimental groups (β-diversity) was computed using unweighted UniFrac and Bray–Curtis distances and their principal coordinate analysis. The effects of putrescine supplementation were assessed by comparing both groups at the last time point (D70). There were significant dissimilarities between the two groups, as shown by the 40% of the variance explained by the principal coordinate axis 1 and the 22% of the variance explained by the principal coordinate axis 2 from unweighted UniFrac distance (*P* < 0.05; Fig. 4B; Supplementary Fig. S3B), as well as the 40% of the variance explained by the principal coordinate axis 1 and 21% of the variance explained by the principal coordinate axis 2 from Bray–Curtis distance (*P* < 0.05; Fig. 4B; Supplementary Fig. S3B).

To detect the effects of putrescine on gut microbiota composition, bacterial relative abundances at the phylum level were analyzed (Fig. 4C; Supplementary Fig. S3C). Putrescine treatment induced a significant increase in the *Firmicutes* to *Bacteroidetes* ratio (vehicle: 1.5 ± 0.2 vs. putrescine: 6.8 ± 2.4 *Firmicutes*/*Bacteroidetes* ratio, *P* < 0.05; Fig. 4C) and a decrease in *Proteobacteria* (vehicle: 0.078% vs. putrescine: 0.007%, relative abundance, *P* < 0.05; Fig. 4C). Additionally, throughout the entire experiment, mice supplemented with putrescine consistently exhibited lower levels of *Enterobacteriaceae* compared with the control group (Supplementary Fig. S4A). Moreover, the relative abundances of *Firmicutes* and *Bacteroidetes* fluctuated throughout the DSS treatment, although significant changes were observed only for *Bacteroidetes* (Supplementary Fig. S4B and S4C).

Within the phylum *Bacteroidetes*, mice that received putrescine showed a decrease in the genera *Alistipes* and *Parabacteroides* and an increase in *Bacteroides* and *Muribaculum*. In the phylum *Firmicutes*, putrescine enhanced the relative abundance of two genera from the *Lachnospiraceae* family, namely, *Limosilactobacillus* and *Anaerotruncus*, while decreasing *UCG-001* and *Ruminococcus gnavus* species group. Within the *Proteobacteria* phylum, the relative abundance of *Escherichia–Shigella* was significantly lower in the putrescine-treated mice (*P* < 0.001; Fig. 4C). Additionally, mice receiving putrescine had augmented the relative abundance of the genus *Enterorhabdus* from the *Actinobacteria* phylum.

### Putrescine induces metabolic shifts linked to DNA repair, cell death, and the acetate pathway

We next investigated whether the compositional differences in microbial taxa between mice receiving the vehicle and those receiving putrescine would result in modifications of predicted microbial functional traits.

We used the Tax4Fun2 tool to infer the metagenomes from 16S rRNA gene analysis and predict the presence of 6,025 Kyoto Encyclopedia of Genes and Genomes orthology terms. Next, we investigated metabolic pathways that have been reported to be involved in promoting or preventing colorectal cancer (Supplementary Fig. S5).

**Figure 5:**
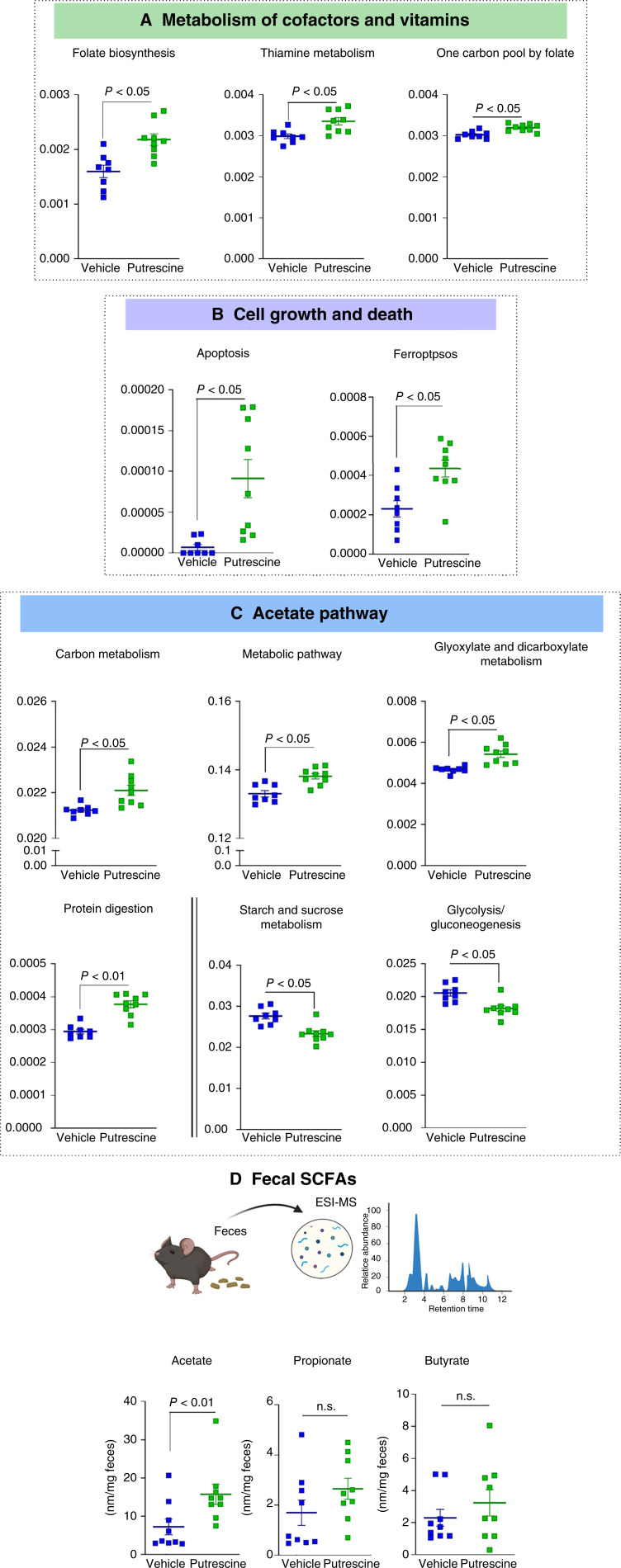
Putrescine supplementation significantly affects gut microbiota functions. Normalized relative abundances of level 3 Kyoto Encyclopedia of Genes and Genomes pathways: (**A**) Metabolism of cofactors and vitamins, (**B**) cell growth and death, and (**C**) acetate production pathways were detected as significantly different by FDR-corrected Kruskal–Wallis sum-rank tests between groups. **D,** Concentration of acetate, propionate, and butyrate per mg feces measured by LC-MS, (*t* test; mean ± SEM) *N* = 8–9 mice. ESI-MS, electrospray-mass spectrometry.

As shown in Fig. 5A, mice given putrescine were predicted to have a higher number of genes implicated in cofactors and vitamins: folate biosynthesis, thiamine metabolism, and one carbon pool by folate, which are involved in DNA repair. In addition, putrescine treatment resulted in the elevated levels of apoptosis- and ferroptosis-related genes involved in programmed cell deaths (Fig. 5B).

As mice receiving putrescine had a higher abundance of SCFA producers from *Lactobacillaceae* (31) and *Lachnospiraceae* (32) families, we further analyzed the SCFA metabolic pathways. Although there were no predicted significant differences in the overall SCFA metabolism pathways between putrescine-treated mice and vehicle-treated mice, there was an increase in the abundance of genes involved in pathways associated with SCFA secretion such as carbon metabolism, metabolic pathway, glyoxylate and dicarboxylate metabolisms, and protein digestion (Fig. 5C). These results were confirmed by directly measuring SCFAs in fecal samples. We found a significant increase in the concentration of acetate in the feces of mice given putrescine (Fig. 5D), which seems to be independent of starch and sucrose metabolism or glycolysis and glycogenosis pathways (Fig. 5C). However, putrescine did not affect the concentration of propionate and butyrate (Fig. 5D). Overall, these data indicate that putrescine induced significant metabolic shifts that are relevant to colorectal cancer development.

### *pks+ E. coli* isolated from a patient with colorectal cancer is sensitive to putrescine

As the EcNC101 (serotype O2:H6/41) strain that was sensitive to putrescine was isolated from mice, we next set out to test whether *pks+ E. coli* strains isolated from human fecal samples could similarly be inhibited by putrescine. We isolated the genotoxic strain *E. coli* CRCmo10. Genomic sequencing followed by bioinformatic analyses predicted that CRCmo10 belonged to serotype O1:H7. A search against the NCBI RefSeq database revealed that the *E. coli* 83-pyelo strain, isolated from the urine of a human patient in 1995 in the United States, is the closest in sequence identity to the CRCmo10 strain, with a Jaccard index of 98.10% and an average nucleotide identity of 99.97%. Interestingly, a TBLASTN analysis comparing the protein sequences of SpeA (arginine decarboxylase, UniProt: P21170) and SpeB (agmatinase, UniProt: 60651), both involved in the production of putrescine from L-arginine (33) from *E. coli* K12, showed a similarity of 99% and 100% with the protein sequences of the CRCmo10 strain, respectively. The genome of strain CRCmo10 also contains genes encoding the putrescine receptor proteins PotFGHI (34) which share a 99% similarity to the sequences from *E. coli* K12 (UniProt: P31133, P31134, P31135, and P0AFL1).

**Figure 6:**
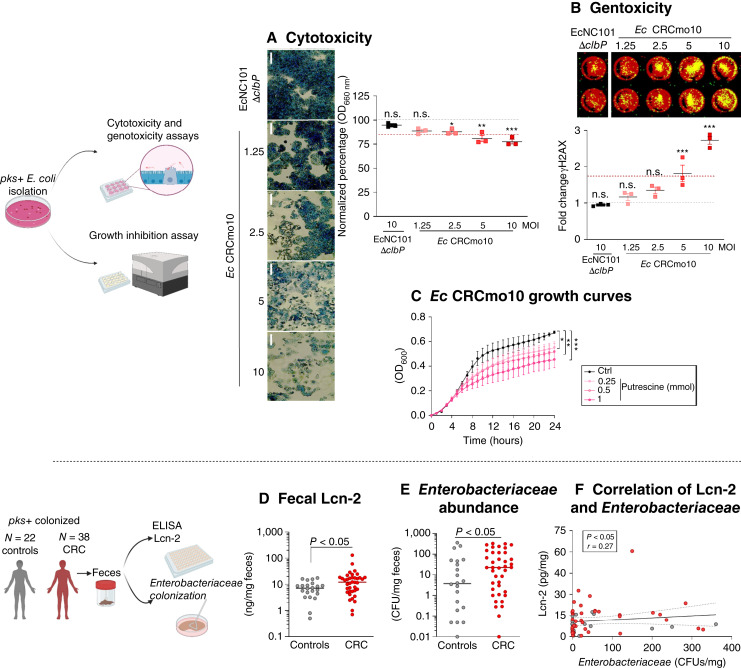
Putrescine lowers the growth of a *pks+ E. coli* strain isolated from a patient with colorectal cancer. **A,** Representative images of methylene blue staining of megalocytosis of HT-29 cells by *E. coli* moCRC10 at 4 hours postinfection (magnification 200×). EcNC101 *ΔclbP* and WT were used as *pks*− and *pks+* control strains, respectively. Quantification of megalocytosis of HT-29 cells. (Upper gray dash line is control values; lower red dash line is EcNC101 values; mean ± SEM, ANOVA *post hoc* Dunnett compared with control; *N* = 3.) **B,** Scan of in-cell Western image showing DNA DSBs with merged detection of total DNA (red, 680 nm) and γ-H2AX (green, 800 nm). γ-H2AX fold induction (analysis was done on the single-colored picture; mean ± SEM, ANOVA *post hoc* Dunnett compared with control; *N* = 3). **C,***E. coli* moCRC10 was grown in the absence or presence of putrescine (mean ± SEM, repeated-measure *t* test; *N* = 5). **D,** Lcn-2 concentration in fecal samples from healthy controls and patients with colorectal cancer. **E,** Abundance of Gram-negative lactose-fermenting strains from the *Escherichia*, *Enterobacteria*, and *Klebsiella* genera [*t* test on log(Y)-transformed data]. **F,** Scatterplot of the association between Lcn-2 and *Enterobacteriaceae*; *N* = 22 healthy individuals/*N* = 38 patients with colorectal cancer. n.s., nonsignificant; *, *P* < 0.05; **, *P* < 0.01; ***, *P* < 0.001.

First, the cytotoxic potential of this newly isolated *pks+ E. coli* strain was evaluated using the megalocytosis assay. At MOI 2.5, 5, and 10, the level of abnormal cell enlargement in human adenocarcinoma HT-29 cells was significantly higher when compared with uninfected cells (Fig. 6A). To confirm its genotoxic potential, we used the in-cell Western assay to quantify DNA DSBs in HT-29 cells infected with *E. coli* CRCmo10. As shown in Fig. 6B, the increasing MOI led to an increase in the DSBs in HT-29 cells as indicated by levels of γ-H2AX, a marker of DNA damage (21).

Next, we evaluated the effect of putrescine supplementation on CRCmo10. As shown in Fig. 6C, 0.25 to 1 mmol/L of putrescine significantly inhibited bacterial growth in a concentration-dependent manner. Additionally, when tested on three other *pks+* strains isolated from human stool samples, putrescine demonstrated significant growth reduction (Supplementary Fig. S6).

Next, fecal Lcn-2 levels were measured in *pks+* healthy individuals and patients with colorectal cancer (ref. 3; Supplementary Table S2). As shown in Fig. 6D, Lcn-2 levels in patients with colorectal cancer were significantly higher compared with those in healthy individuals (controls: 7.8 ± 1.0 vs. colorectal cancer: 16.3 3.4 ng/mg feces, *P* < 0.036). In addition, patients with colorectal cancer had a higher abundance of *Enterobacteriaceae* compared with healthy controls as determined by CFU counts (controls: 47 ± 20 vs. colorectal cancer: 104 ± 37 CFUs, *P* < 0.028; Fig. 6E), and fecal Lcn-2 levels positively correlated with *Enterobacteriaceae* abundance (Fig. 6F).

These data demonstrate that genotoxic *pks+ E. coli* strains isolated from patients with colorectal cancer are also sensitive to the presence of putrescine, which significantly inhibits their growth.

## Discussion

Colibactin-producing bacteria are present in 4.35% to 68% of both healthy individuals and patients with colorectal cancer (35, 36). This study highlights the potential properties of putrescine supplementation in inhibiting the expansion of *pks+ E. coli* strains and colorectal cancer development in a mouse model of colorectal cancer.

### Putrescine effects on growth and expansion of *pks+ E. coli*, tumor development, and inflammation

In this study, we show that putrescine generated by EcN contributes to the inhibition of EcNC101 growth. Putrescine is produced by bacteria belonging to the phyla Acidobacteria, Bacteroidetes, Fusobacteriota, and mainly Proteobacteria (37). For instance, *Bacteroides thetaiotaomicron* and *Fusobacterium varium* are able to produce putrescine and subsequent polyamines in the cecum of germ-free rats (38). Here, we show that EcN produces putrescine through the ODC pathway. Polyamines are formed by agmatine, or ornithine and arginine. The primary secretory pathway is through ODC which generates putrescine that is converted to spermidine and, ultimately, spermine by S-adenosylmethionine decarboxylase 1 enzymes (39). Commensal bacteria primarily use the ODC pathway to produce putrescine, whereas pathogenic bacteria preferentially use the agmatinase pathway (37). The observed growth suppression effect of putrescine on the murine strain EcNC101 and the colorectal cancer–-isolated *pks+ E. coli* strain may be due to the positively charged potential of the molecule, which affects protein structure, function, interaction, and localization (40) and thus reduces the growth of pH-sensitive *E. coli* strains such as EcK12 (41).

We show that EcNC101 colonization enhanced the number and size of colonic tumors in the AOM/DSS mouse model. Similar findings were reported when *E. coli* strains SP15 (42) and CCR20, isolated from human colonic tumors (2), were used in the AOM/DSS mouse model. Additionally, we found that body weight loss and colon length shortening, caused by inflammation (26), were independent of colibactin. This could be explained by the fact that the resulting inflammation following colonization with EcNC101 WT or a colibactin-deficient strain was similar (43).

Although our study does not exclude the possibility that EcN may produce other potential postbiotics that could affect EcNC101 growth, in this study, we show that putrescine supplementation was sufficient to significantly lower tumorigenicity in the AOM/DSS mouse model. Furthermore, our results on the protective effect of putrescine against weight loss and colonic shortening are similar to those previously shown against the effects of DSS treatment in a mouse model of colitis (44).

However, in the *Apc*^*Min/+*^ mouse model, putrescine was shown to reduce the anticarcinogenic activity of sulindac, an NSAID (45). In *Apc*^*Min/+*^ mice, ODC activity is elevated because of alterations in the *Apc* gene and results in the overproduction of polyamines and a hyperproliferative phenotype (46), which could explain the adverse effect of putrescine supplementation in the *Apc*^*Min/+*^ mouse model. The AOM/DSS mouse model has the advantage to mimic colitis-associated colorectal cancer, seen often in patients with inflammatory bowel disease, with flares of inflammation and the development of sporadic tumors, with inflammation playing a major role (28).

We found that putrescine had anti-inflammatory properties by diminishing TNFα, IL6, and Lcn-2 levels, corroborating with similar results reported in an LPS-induced mucosal atrophy in weanling piglets (47) and in azure-winged magpies (48). Another study reported that putrescine generated by gut microbes alleviated colitis in mice by stimulating polarization in M2 macrophages (44). Similarly, our results confirm the fact that gut inflammation is higher in patients with sporadic colorectal cancer compared with healthy controls, which may lead to a leaky gut and result in systemic inflammation (49). Bacteria from the *Enterobacteriaceae* family thrive in an inflammatory gut environment with increased oxygen in the intestinal lumen due to the hypoxic state of the intestinal mucosa and increased nitrogen sources (30). The genotoxic strains of *pks+ E. coli* inducing DSBs are therefore part of the bacteria from the gut microbiota that expand during inflammation and could enhance the formation of tumors in an inflamed gut.

### Putrescine-induced shifts in gut microbiota composition and function

Putrescine supplementation increased α-diversity of the gut microbiome, which is beneficial because a low α-diversity is associated with a more advanced stage of colorectal cancer (50). There is limited research available on putrescine supplementation and its influence on the gut microbiota. Harrold and colleagues (48) showed an increased abundance of the genera *Bacillus* and *Lactobacillus* in azure-winged magpies that received dietary supplementation with putrescine. Overall, we found that putrescine increased the *Firmicutes*/*Bacteroidetes* ratio, and a higher Firmicutes/Bacteroidetes ratio is linked to better survival in patients with colorectal cancer (51). In the phylum *Firmicutes*, we found an increase in the *Limosilactobacillus* genus in which *Limosilactobacillus fermentum* has been reported to produce peptidoglycan with anti-proliferative properties (52) and *Limosilactobacillus reuteri* has been shown to suppress colorectal tumorigenesis (53). *Lachnospiraceae* are known to produce SCFAs and to play a beneficial role in intestinal health. Nevertheless, their role in inflammatory bowel disease and colorectal cancer has been questioned (32) as *Ruminococcus gnavus* possesses mucolytic properties that are potentially harmful in the context of inflammatory bowel disease (54). Putrescine supplementation decreased the relative abundance of the genus *Alistipes*, which has been associated with colorectal cancer development (55). *Alistipes* were increased in fecal samples from patients with advanced stage colorectal cancer (56), and *Alistipes finegoldii* was shown to promote colorectal cancer through activation of the IL6/STAT 3 pathway (57). *Escherichia–Shigella* genus, which is expanded in patients with colorectal cancer (58) and is a known pathobiont involved in colorectal cancer development (59), was lowered by putrescine supplementation.

The modification of the gut microbiota after putrescine supplementation was associated with an increase in genes related to DNA repair and programmed cell death. In particular, folate both maintains genomic stability by regulating DNA biosynthesis, repair, and methylation (60), as well as controls cancer cell proliferation (61). Thiamine has been shown to act as an antioxidant by scavenging reactive oxygen species and thus preventing DNA damage (62). With regard to cell death, abnormalities in apoptotic function have been established as a hallmark of colorectal cancer (63) as programmed cell death is required to preserve genome integrity (64). In this aspect, reducing Lcn-2 levels by putrescine could favor genomic stability, as Lcn-2 has been shown to inhibit ferroptosis (65), an iron-dependent, nonapoptotic regulated cell death characterized by the accumulation of lipid reactive oxygen species (66). Additionally, we report an increase in acetate levels in mice that received putrescine compared with mice treated with vehicle. Interestingly, lower acetate levels have been reported in individuals at higher risk of developing colorectal cancer as well as in patients with colorectal cancer (67), as acetate has been shown to induce growth arrest (68) and apoptosis (69) in colon cancer cells.

In conclusion, we showed that putrescine limits *pks+ E. coli* expansion and lowers inflammation in the AOM/DSS mouse model of colorectal cancer, resulting in decreased tumor burden and inflammation (Supplementary Fig. S7). Our findings suggest that probiotic-derived metabolites can be used as an alternative to live bacteria in individuals at risk of developing colorectal cancer due to the presence of *pks+* bacteria in their colon. Therefore, consumption of healthy foods rich in putrescine may present a potential approach to reduce the risk of colorectal cancer among *pks+* individuals.

## Supplementary Material

Figure S1 Figure S1 shows E. coli metabolome and ornithine decarboxylase activity

Figure S2 Figure S2 shows the effects of putrescine supplementation on cell proliferation

Figure S3 Figure S3 shows the effect of putrescine on gut microbiota

Figure S4 Figure S4 shows gut microbiota changes after DSS-induced flares

Figure S5 Figure S5 shows the effect of putrescine on gut microbiota functions

Figure S6 Figure S6 shows growth of pks+ E. coli strains isolated from human samples

Figure S7 Figure S7. Putrescine lowers inflammation, inhibits pks+ E. coli growth, and reduces tumor development.

Table S1 Table S1 shows cohort characteristics

Table S2 Table S2 shows primers used
