# Supplementary material for: Putrescine Supplementation Limits the Expansion of pks+ Escherichia coli and Tumor Development in the Colon
Source: Cancer Res Commun. 2024 Jul 22;4(7):1777–92. doi: 10.1158/2767-9764.CRC-23-0355 (PMC11261243; doi:10.1158/2767-9764.CRC-23-0355)
Supplement: Figure S1 — shows E. coli metabolome and ornithine decarboxylase activity [file crc-23-0355_figure_s1_supps1.docx]

**
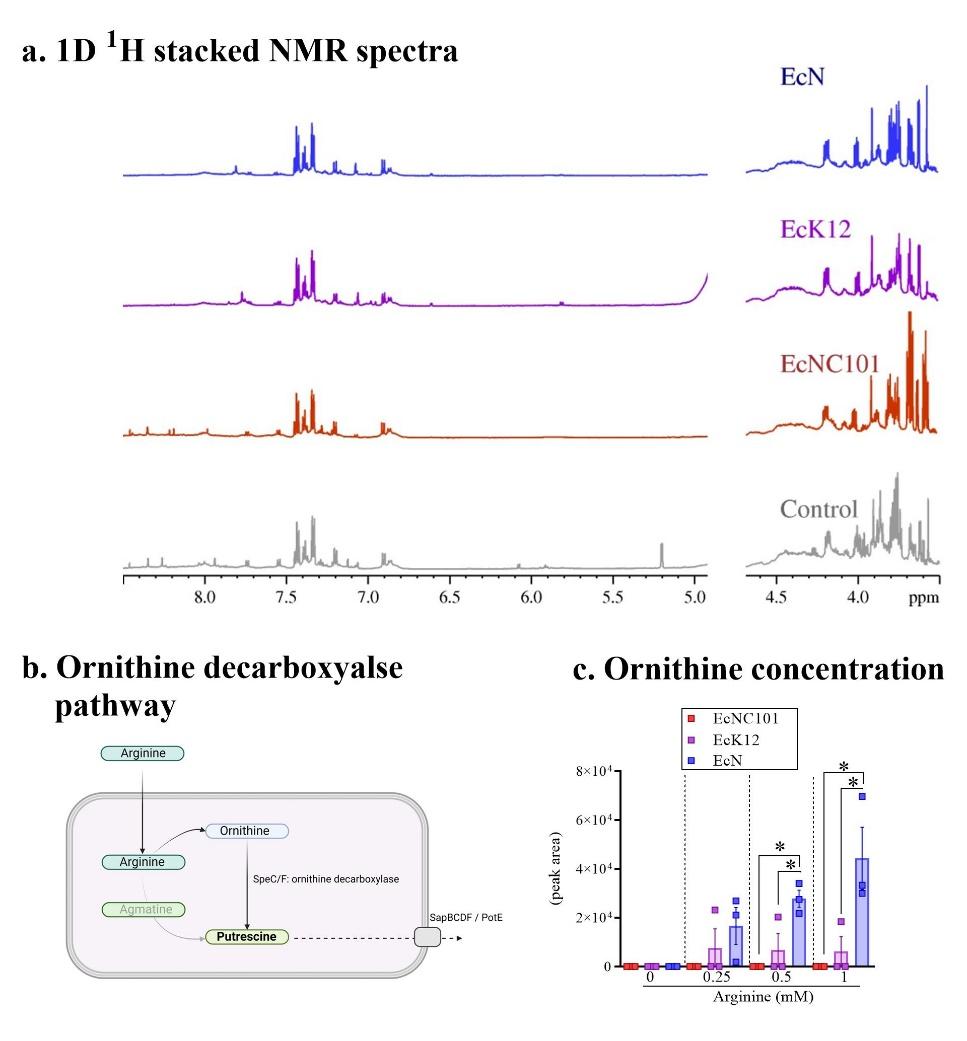
**

**Figure S1**. ***E. coli* metabolome and ornithine decarboxylase activity**. (**a**) 1D ^1^H NMR spectra of supernatants from EcN, EcK12, EcNC101, and control (lysogeny broth; LB). Spectra are from 3.5 to 8.5 ppm and water signal from 4.7 to 4.9 ppm is deleted. (**b**) Schematic of ODC pathway. (**c**) Ornithine to arginine ratio from supernatants of EcNC101, EcK12 and EcN grew in minimal medium (M9) supplemented with or without increasing concentrations of arginine (ANOVA, compared to 0.2; N = 3). **P* < 0.05.
