# Supplementary material for: Putrescine Supplementation Limits the Expansion of pks+ Escherichia coli and Tumor Development in the Colon
Source: Cancer Res Commun. 2024 Jul 22;4(7):1777–92. doi: 10.1158/2767-9764.CRC-23-0355 (PMC11261243; doi:10.1158/2767-9764.CRC-23-0355)
Supplement: Figure S2 — shows the effects of putrescine supplementation on cell proliferation [file crc-23-0355_figure_s2_supps2.docx]

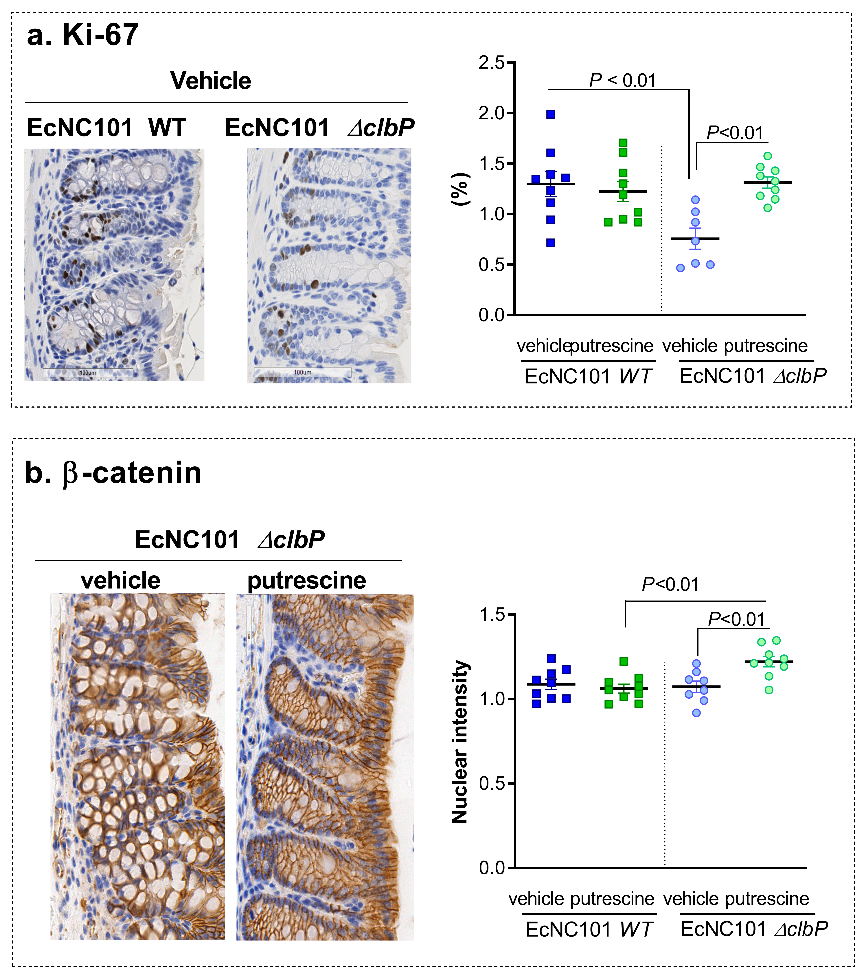


**Figure S2. Effects of putrescine supplementation on cell proliferation markers in the AOM/DSS CRC mouse model.** (**a**) Quantification of Ki-67-positive cells per healthy mucosa site. (**b**) Quantification of β-catenin-positive cells per healthy mucosa site. ANOVA, post-hoc Tukey; N = 8-9 per group.
