# Supplementary material for: Putrescine Supplementation Limits the Expansion of pks+ Escherichia coli and Tumor Development in the Colon
Source: Cancer Res Commun. 2024 Jul 22;4(7):1777–92. doi: 10.1158/2767-9764.CRC-23-0355 (PMC11261243; doi:10.1158/2767-9764.CRC-23-0355)
Supplement: Figure S3 — shows the effect of putrescine on gut microbiota [file crc-23-0355_figure_s3_supps3.docx]

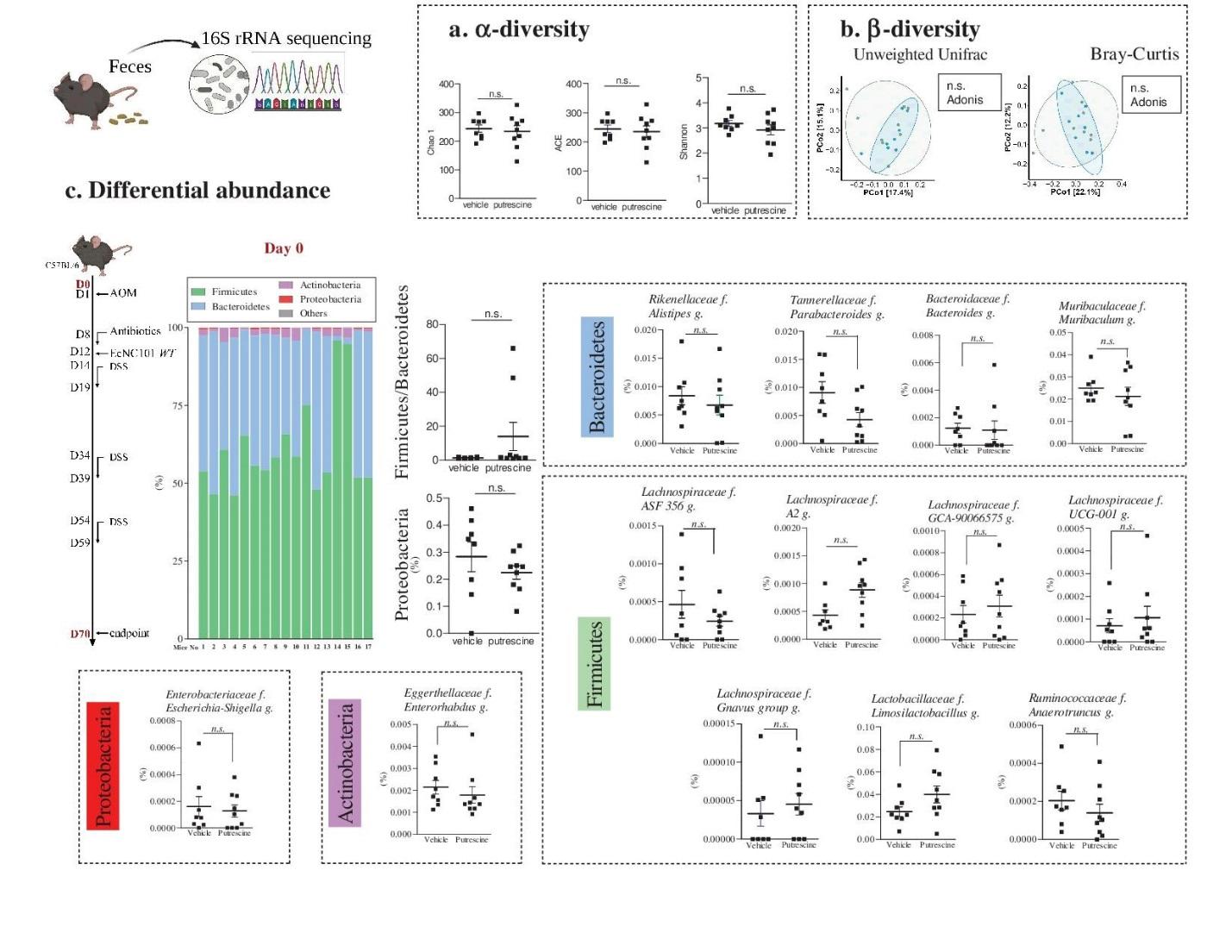


**Figure S3. Gut microbiota features affected by putrescine supplementation were similar at D0.** (**a**) Chao1, ACE and Shannon indexes at day 0 (mean ± SEM, *t*-test). (**b**) Principal coordinates analysis (PCoA) of the unweighted UniFrac and Bray-curtis distances. (**c**) Differential bacterial abundance at the phylum and genus levels (mean ± SEM, *fdr* corrected). Bar graphs of relative abundances at the phylum level at day 0. Each stacked bar represents a single mouse. N = 17 mice.
