# Supplementary material for: Putrescine Supplementation Limits the Expansion of pks+ Escherichia coli and Tumor Development in the Colon
Source: Cancer Res Commun. 2024 Jul 22;4(7):1777–92. doi: 10.1158/2767-9764.CRC-23-0355 (PMC11261243; doi:10.1158/2767-9764.CRC-23-0355)
Supplement: Figure S4 — shows gut microbiota changes after DSS-induced flares [file crc-23-0355_figure_s4_supps4.docx]

**
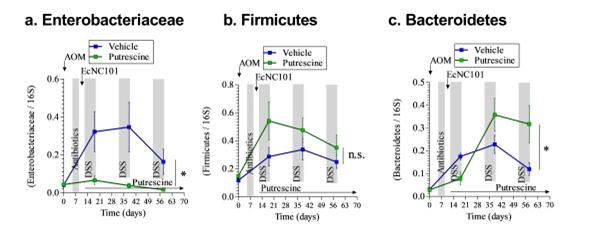
Figure S4. Gut microbiota changes after DSS-induced flares.** (**a**) Enterobacteriaceae, (**b**) Firmicutes, and (**c**) Bacteroidetes abundance following DSS-induced flares (mean ± SEM, repeated-measure ANOVA). N = 9 per group.
