# Supplementary material for: Putrescine Supplementation Limits the Expansion of pks+ Escherichia coli and Tumor Development in the Colon
Source: Cancer Res Commun. 2024 Jul 22;4(7):1777–92. doi: 10.1158/2767-9764.CRC-23-0355 (PMC11261243; doi:10.1158/2767-9764.CRC-23-0355)
Supplement: Figure S5 — shows the effect of putrescine on gut microbiota functions [file crc-23-0355_figure_s5_supps5.docx]

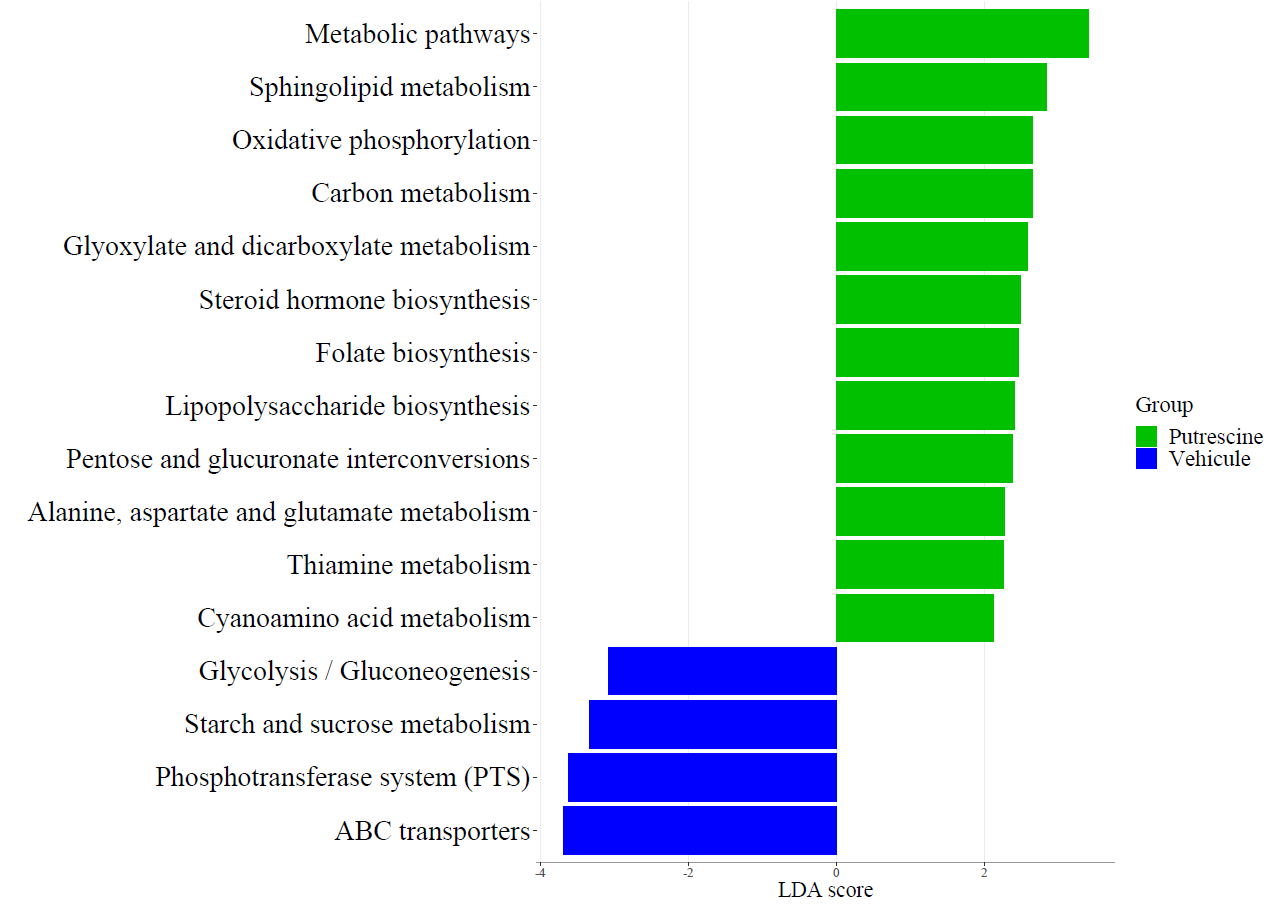


**Figure S5**. **Putrescine supplementation significantly affects gut microbiota functions.** Comparison of relative abundance of functional level 3 pathways between mice supplemented with putrescine (green) or vehicle (blue) using Linear discriminant analysis effect size (LEfSe) analysis generated from the Tax4Fun2 pipeline. A number of 12 KEGG pathways are significantly enriched in mice under putrescine supplementation and 4 KEGG pathways are enriched in vehicle-treatedmice. Data show differences in predicted bacterial metabolic function between mice supplemented with putrescine or vehicle.
