# Supplementary material for: Putrescine Supplementation Limits the Expansion of pks+ Escherichia coli and Tumor Development in the Colon
Source: Cancer Res Commun. 2024 Jul 22;4(7):1777–92. doi: 10.1158/2767-9764.CRC-23-0355 (PMC11261243; doi:10.1158/2767-9764.CRC-23-0355)
Supplement: Figure S6 — shows growth of pks+ E. coli strains isolated from human samples [file crc-23-0355_figure_s6_supps6.docx]

**
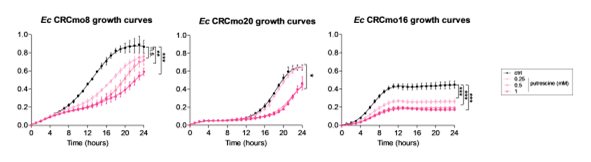
Figure S6. Putrescine lowers the growth of pks*+ E. coli* strains isolated from human stool samples.** *Pks+ E. coli* strains were grown in the absence or presence of putrescine (mean ±SEM, repeated-measure *t*-test; N=5).
