# Supplementary material for: Putrescine Supplementation Limits the Expansion of pks+ Escherichia coli and Tumor Development in the Colon
Source: Cancer Res Commun. 2024 Jul 22;4(7):1777–92. doi: 10.1158/2767-9764.CRC-23-0355 (PMC11261243; doi:10.1158/2767-9764.CRC-23-0355)
Supplement: Figure S7 — Putrescine lowers inflammation, inhibits pks+ E. coli growth, and reduces tumor development. [file crc-23-0355_figure_s7_supps7.docx]

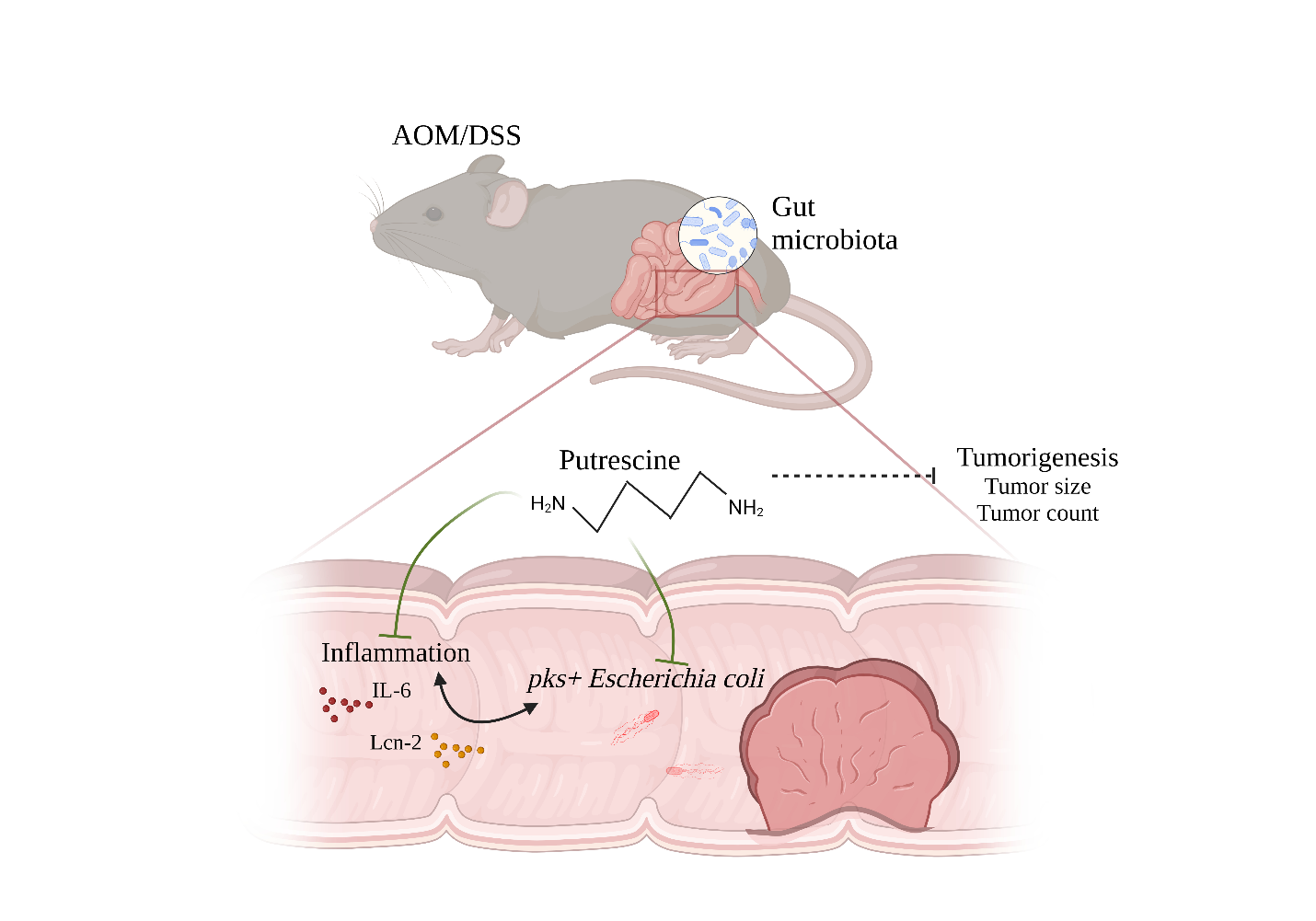


**Figure S7. Putrescine lowers inflammation, inhibits pks+ E. coli growth, and reduces tumor development.** Schematic diagram of the effect of putrescine on inflammation, *pks+ E. coli* expansion and tumor burden in the AOM/DSS mouse model of CRC.
