# Supplementary material for: Putrescine Supplementation Limits the Expansion of pks+ Escherichia coli and Tumor Development in the Colon
Source: Cancer Res Commun. 2024 Jul 22;4(7):1777–92. doi: 10.1158/2767-9764.CRC-23-0355 (PMC11261243; doi:10.1158/2767-9764.CRC-23-0355)
Supplement: Table S1 — shows cohort characteristics [file crc-23-0355_table_s1_suppst1.docx]

**Table S1. Cohort characteristics of the *pks+* individuals**

|  | Healthy controls (N=22) | CRC patients (N=39) | *p*-value |
| --- | --- | --- | --- |
| Age (median, [range]) | 59, [36-71] | 68, [41-79] | *0.0013* |
| Sex, n (%)  Female  Male | 9 (41)  13 (59) | 11 (28)  28 (72) | *0.3101* |
| Location, n (%)  proximal  distal | n.a.  n.a. | 13 (33)  26 (67) |  |
| Stage, n (%)  I  II  III  IV | n.a.  n.a.  n.a.  n.a. | 5 (13)  13 (33)  17 (44)  4 (10) |  |

Age: t-test

Sex: Fisher test

Abbreviation: CRC, colorectal cancer; n.a., not applicable.
