# Supplementary material for: Putrescine Supplementation Limits the Expansion of pks+ Escherichia coli and Tumor Development in the Colon
Source: Cancer Res Commun. 2024 Jul 22;4(7):1777–92. doi: 10.1158/2767-9764.CRC-23-0355 (PMC11261243; doi:10.1158/2767-9764.CRC-23-0355)
Supplement: Table S2 — shows primers used [file crc-23-0355_table_s2_suppst2.docx]

**Table S2. Primers**

| **Name** | **Sequence** |
| --- | --- |
| Entero_Fw | CATTGACGTTACCCGCAGAAGAA |
| Entero_Rv | CGCTTGCACCCTCCGTATTA |
| Firm_Fw | ACCCGCGTCTGATTAGCTAGTT |
| Firm_Rv | CCTCTCAGGCCGGCTACTG |
| Bacte_Fw | CCTWCGATGGATAGGGGTT |
| Bacte_Rv | TCCCCAGGTGGAATACTTAACG |
